# Synergistic PVA/zein electrospun nanofiber array with curcumin-alizarin: a water-resistant, sensitive colorimetric platform for graded pork freshness monitoring

**DOI:** 10.1016/j.fochx.2026.104591

**Published:** 2026-10-07

**Authors:** Bingcan Liu, Siling Zhang, Honghui Geng, Can Hu, An Zhou, Zeyu Wu

**Affiliations:** a Engineering Research Center of Bio-Process of Ministry of Education, School of Food and Biological Engineering, Hefei University of Technology, Hefei 230601, China; b School of Materials Science and Engineering, Hunan Institute of Technology, Hengyang 421002, China; c The Experimental Research Center, Anhui University of Chinese Medicine, Hefei 230038, China; d Intelligent Manufacturing Institute, Hefei University of Technology, Hefei 230051,China

**Keywords:** Electrospinning, Intelligent packaging, Dual-pigment indicator, Natural pigments, Volatile amines, Meat spoilage

## Abstract

In this work, a synergistic polyvinyl alcohol (PVA)/zein electrospun nanofiber array was fabricated using curcumin and alizarin as dual responsive pigments for visual pork freshness monitoring. The water solubility of the PVA/zein composite reduced from 100.00% to 31.74%–35.42% (*P* < 0.05), exhibiting remarkably enhanced water resistance. The dual-pigment array displayed continuous color transitions from pH 4.0 to 11.0 and detected ammonia and dimethylamine at 1 ppm, whereas alizarin-only films needed 5 ppm. The total color difference (*ΔE*) of the array showed strong linear correlations with thiobarbituric acid reactive substances (TBARS, *r* = 0.98), pH (*r* = 0.95), and total volatile basic nitrogen (TVB-N, *r* = 0.98) in pork during refrigerated storage. Notably, the array enabled visual grading of pork freshness (fresh/sub-fresh/spoiled) and exhibited excellent color stability under ambient and refrigerated conditions. This study presents an eco-friendly and scalable intelligent packaging platform with great potential for real-time, on-site food freshness assessment.

## Highlights

• Fabrication of indicator array based on varying proportions of curcumin and alizarin.
• Composite films exhibited a lower water solubility compared with pure PVA films.
• The detection limit of indicator array for volatile amine gases was down to 1 ppm.
• Color alterations of the array strongly correlated with pork spoilage indexes.

## Graphical abstract

In this study, a colorimetric array based on polyvinyl alcohol-zein composite substrates and varying ratios of curcumin and alizarin as responsive materials was fabricated via electrospinning for monitoring pork freshness during refrigeration.

**Figure:**
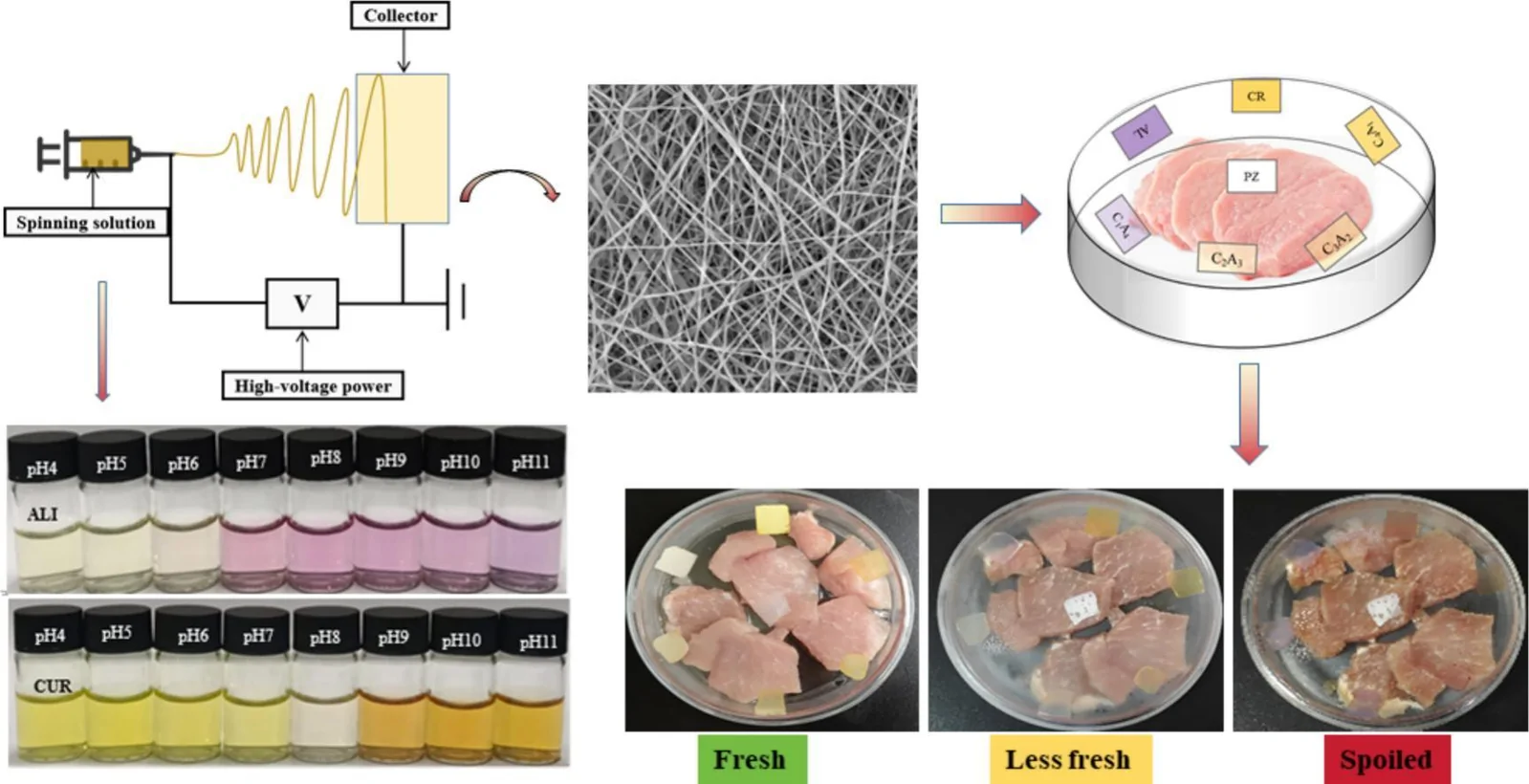

## 1 Introduction

Pork is a staple in the global meat market due to its affordability and consumer appeal. Projections by the Organization for Economic Co-operation and Development (OECD) indicate that global pork consumption will reach 129 million metric tons by 2032, representing one-third of the projected total growth in global meat consumption (OECD/FAO, 2022). However, the high lipid and protein content of pork, combined with its storage in high-humidity environments, renders it prone to microbial spoilage, leading to degradation of nutritional value and palatability. This not only poses a health risk to consumers, but also causes resource waste and economic losses. Traditionally, pork freshness has been evaluated by consumers through subjective sensory attributes like color and odor. However, these approaches lack reliability due to inconsistent standardization. Moreover, conventional freshness detection methods are facing challenges, such as high cost, the need for skilled personnel, and complex sample pretreatment procedures (Yu et al., 2024). Therefore, it is essential to establish a swift and convenient detection method capable of assessing meat freshness in real time and suitable for application in both domestic and retail environments.

Smart labels have emerged as innovative tools for real-time food quality monitoring, responding to environmental shifts such as temperature and gas composition (Chen et al., 2023; Gong et al., 2025). During meat storage, microbial and enzymatic activity generates volatile compounds within packaging, altering internal pH values. Freshness indicator labels utilize pH-sensitive pigments to assess the status of meat based on color variations. Although synthetic pigments are stable and cost-effective, concerns about their health and environmental risks have led to a preference for non-toxic natural pigments (Charoensit et al., 2021).

Curcumin (Cur), as a natural polyphenolic compound extracted from the rhizome of turmeric, possesses notable antibacterial, antioxidant, and pH-sensitive characteristics (El-Desoky et al., 2020; El-Desoky et al., 2021; El-Desoky et al., 2022). In acidic environments, it displays a yellow hue, whereas in alkaline conditions, it shifts to red. Similarly, alizarin, also known as 1,2-dihydroxyanthraquinone, is primarily derived from the roots of the madder plant. As an anthraquinone compound, alizarin is sensitive to pH fluctuations, exhibiting color transitions from yellow in acidic conditions to purple in alkaline environments (Ezati et al., 2019). Ezati and Choi (Choi et al., 2024; Ezati & Rhim, 2020) have demonstrated that both Cur and alizarin effectively function as freshness sensors in smart labels for meat products. Nevertheless, the application of a single natural pigment presents certain limitations, including a narrow pH response range and limited discoloration. Combining multiple pigments or employing colorimetric arrays can enhance color diversity, broaden detection ranges, and improve sensitivity and accuracy (Wang et al., 2022).

Polyvinyl alcohol (PVA), a biodegradable polymer widely used in packaging, offers excellent film-forming and mechanical properties. However, its susceptibility to high water vapor permeability leads to swelling or even dissolution in humid environments, destroying membrane integrity (He et al., 2022; Huang et al., 2026). To mitigate this problem, methods including aldehyde gas crosslinking and blending have been developed (Luo et al., 2024; Xiao et al., 2023). While gas crosslinking effectively blocks excess hydroxyl groups, it involves the utilization of hazardous reagents. Blending, by contrast, offers a safer and more sustainable alternative. Zein, a hydrophobic protein rich in nonpolar amino acids, possesses good alcohol solubility and hydrophobicity, along with antimicrobial resistance and safety (Ren et al., 2022; Vogt et al., 2018). Although zein-based films exhibit excellent barrier properties, their poor mechanical properties restrict the applicability in food packaging (Xiao et al., 2022). According to previous research performed by Senna (Senna et al., 2010), PVA/zein composites showed improved mechanical and water-resistant properties, yet existing smart indicators still face key challenges. For example, single PVA films lacked stability in humid conditions, single natural pigments exhibited a narrow pH response and weak color contrast, and most indicators offered only qualitative changes without clear freshness grading.

Electrospinning is a method used to prepare nanofibers. In this process, a charged liquid forms a Taylor cone within an electric field, from which a charged jet is expelled. As this jet moves, it undergoes whipping and stretching, ultimately forming solid fibers that are collected on a reel (Xue et al., 2019). The resulting nanofiber mat is characterized by a high surface-to-volume ratio and significant porosity, rendering it an ideal candidate for intelligent packaging applications (Aghababaei et al., 2024). Importantly, electrospinning avoids high-temperature reactions, thereby preserving the stability and efficacy of incorporated pH indicators.

To address these challenges, a synergistic PVA/zein electrospun nanofiber array embedded with Cur and alizarin as dual responsive materials was developed in this study. A colorimetric array using Cur and alizarin blended at varying ratios was designed to improve detection accuracy. The PVA/zein composite was electrospun as a polymer carrier to enhance structural stability under humid conditions. The array performance was systematically characterized, and its color response, sensitivity, and selectivity toward volatile amines were evaluated. The efficacy of the indicator array for monitoring freshness during refrigerated storage was validated with pork tenderloin as a model.

## 2 Materials and methods

### 2.1 Materials and reagents

PVA (1788, alcoholysis 87%–89%) and zein were obtained from Aladdin Biochemical Technology Co., Ltd. (Shanghai, China). Acetic acid (guaranteed reagent) and ammonium hydroxide solution (25–28 wt%) were purchased from Sinopharm Chemical Reagent Co., Ltd. (Shanghai, China). Alizarin, Cur, dimethylamine solution (40% in H_2_O), trimethylamine solution (30% in H_2_O), trichloroacetic acid (TCA), 4,6-dihydroxy-2-mercaptopyrimidine (TBA, 98%) and other chemicals were acquired from Macklin Biochemical Technology Co., Ltd. (Shanghai, China). All reagents not specifically mentioned were of analytical grade.

### 2.2 Preparation of nanofiber film

The zein solution was formulated in accordance with the method described by Li (Li et al., 2020), by dissolving 10 g of zein powder in 100 mL of a solvent mixture consisting of acetic acid and distilled water (4:1, V/V) at 25 °C under magnetic stirring at 300 rpm for 4 h. After that, a PVA solution was prepared by dissolving 10 g of PVA powder in 100 mL of a mixture of acetic acid and distilled water (4:1, V/V) and stirring at 90 °C (300 rpm, 2 h) until complete dissolution. The concentrations of both solutions were 10% (w/v), and both were cooled to 25 °C before blending. Based on preliminary test results, the zein and PVA solutions were blended uniformly in a 7:3 volume ratio. Then, 3 mg of mixed pigments consisting of Cur and alizarin was added to the above mixture at various mass ratios of 5:0, 4:1, 3:2, 2:3, 1:4, and 0:5. The spinning solution was degassed by ultrasound treatment for 5 min to remove bubbles. The prepared spinning solution was filled into a 5 mL syringe equipped with a 22-gauge needle. The electrostatic spinning equipment (YFSP-T, Yunfan Technology, China) was operated under the following conditions: an applied voltage of 16 kV, a feed rate of 0.5 mL/h, and a collecting distance of 10 cm. The environmental temperature was set at 35 °C with humidity levels ranging from 25% to 30%. The resultant nanofibers were designated as CR, C_4_A_1_, C_3_A_2_, C_2_A_3_, C_1_A_4_, and AL, according to their pigment content. Furthermore, the PVA-zein composite film without pigments and the pure PVA film without pigments were designated PZ and PA, respectively.

### 2.3 UV–vis spectra of pigments at different pH values

The UV–vis spectra of Cur and alizarin were obtained at various pH values, between 4.0 and 11.0, using a UV-spectrophotometer (UV-1800PC, AOELAB, China) across a wavelength range of 250 to 700 nm. Simultaneously, the color responses of the mixed pigment solutions at these differing pH values were recorded with a smartphone.

### 2.4 Characterization of nanofiber films

#### 2.4.1 Scanning electron microscopy (SEM)

Referring to Liu's method (Liu et al., 2020), the samples were sectioned into squares measuring 5 mm × 5 mm and adhered to the sample table with double-sided conductive adhesive. Then, the samples were sprayed with gold under vacuum for 60 s. A scanning electron microscope (EM-30+, COXEM, Korea) was used to observe the surface morphologies of the samples at a magnification of 10,000×. To evaluate the distribution of nanofiber diameters, measurements were taken at 100 randomly chosen sites within the SEM images using ImageJ software (NIH, Bethesda, USA).

#### 2.4.2 Fourier transform infrared spectroscopy (FTIR)

The interactions among the film components were characterized using a Fourier transform infrared spectrometer (Spectrum Two, PerkinElmer, USA) with a resolution of 4 cm^−1^ across the frequency range of 4000 to 400 cm^−1^.

#### 2.4.3 Thickness and mechanical properties

According to Huang et al. (2023), with slight modifications, the thickness of the samples was estimated at five randomly selected sites by a thickness gauge (DLX-chg30, DELIXI, China), which possessed an accuracy of 0.001 mm. The samples were shaped into a dumbbell configuration, using a mold with specific dimensions, that is, a total length of 50 mm, an end width of 8 mm, and a central width of 4 mm. The sample mechanical characteristics were evaluated with a texture analyzer (XT-PLUSC, Stable Micro Systems, UK). During the test, the initial distance between the chucking was set at 30 mm, with a movement rate of 3.3 mm/min.

#### 2.4.4 Moisture content, water solubility and swelling index

The water sensitivity of films was assessed following the methodology established by Yue et al. (2024). Briefly, the samples were sectioned into squares (25 mm × 25 mm), and their initial mass (M_0_) was recorded. Then, the samples were heated at 105 °C until a stable weight (M_1_) was reached. After drying, the samples were divided into two groups. The first group was fully immersed in 20 mL of distilled water at 25 °C for 24 h. Following this immersion period, the residues were taken out, dried again at 105 °C until a constant weight was achieved and recorded as M_2_. In the second group, after immersion in 20 mL of distilled water at 25 °C for 30 min, the samples were wiped using filter paper to remove the surface moisture, and then their weight was measured again and denoted as M_3_. The moisture content (MC), water solubility (WS) and swelling index (SI) were calculated by the following Eqs. (1), (2), (3), respectively:

(1) $$\mathrm{MC}=\frac{M_{0}-M_{1}}{M_{0}}\times 100\%$$

(2) $$\mathrm{WS}=\frac{M_{1}-M_{2}}{M_{1}}\times 100\%$$

(3) $$\mathrm{SI}=\frac{M_{1}-M_{3}}{M_{1}}\times 100\%$$

#### 2.4.5 Water vapor permeability (WVP)

The WVP of films was assessed utilizing the gravimetric method (ASTM E96-95) (ASTM, 2024) with certain modifications. Initially, 20 mL of distilled water was introduced into a cup, which was sealed with the film and placed in a drying apparatus containing anhydrous silica gel. The weight of the cup was measured at 12-h intervals until a stable weight was obtained. WVP was calculated by the following Eq. (4):

(4) $$\mathrm{WVP}=\frac{∆m\times d}{∆t\times A\times ∆P}$$

where Δ *m* /Δ *t* was the rate of water loss per unit time (g/h), *A* was the film permeation area (m^2^), *d* was the film thickness (m), and Δ*P* was the difference in water vapor pressure across the two sides of the film (Pa).

### 2.5 Sensitivity of nanofiber films to different volatile amines

Referring to the approach of Zhai et al. (2020), samples (10 mm × 10 mm) were affixed to the inner surface of a glass dish, and then inverted onto a flat glass surface to form a sealed reaction chamber. The initial color parameters of the films, including *L*, a, and b values, were recorded by using a colorimeter (NH310, 3nh, China). Dimethylamine (DMA), trimethylamine (TMA), and ammonia were chosen as test substances, and varying concentrations (1, 5, 10, 20 and 40 ppm) of test substances were injected into the reaction chamber. After 20 min, the color parameters were recorded again. The total color difference (∆E) was determined by Eq. (5), while the concentration of test substances in the reaction chamber was calculated using Eq. (6):

(5) $$∆E=\sqrt{{\left(L_{0}-L_{1}\right)}^{2}+{\left(a_{0}-a_{1}\right)}^{2}+{\left(b_{0}-b_{1}\right)}^{2}}$$

(6) $$C=\frac{\rho V_{1}W}{MV_{0}}$$

where *L*_*0*_, *a*_*0*_ and *b*_*0*_ represented the initial color parameters of film samples, and *L*_*1*_, *a*_*1*_ and *b*_*1*_ were the color parameters of samples after reaction; *C* was the test substance concentration in the reaction chamber (mol/L), *ρ* was the density of the test substance solution (g/mL), *V*_*1*_ was the volume of test substance injection (mL), *W* was the mass fraction of the test substance in the solution; *M* was the molar mass of the test substance (g/mol); and *V*_*0*_ was the volume of the reaction chamber (L).

### 2.6 Color stability of nanofiber films

The film samples were stored at 4 and 25 °C, respectively, for 10 d, and the color parameters of the films were recorded every 2 days under consistent lighting conditions. The color parameters measured on day 0 were taken as the initial values. The variations in the ΔE values of the films throughout the storage period were employed to evaluate the inherent color stability of the films.

### 2.7 Application for pork freshness monitoring

#### 2.7.1 Pork freshness monitoring trial

Pork tenderloin samples (chilled, never frozen, without marinade or added preservatives) were procured from a single retail batch with the same sell-by date at a local supermarket (Hefei, China) and transported to the laboratory in insulated containers at 4 °C within 0.5 h. Only portions with a normal pinkish-red color, free of surface discolouration, off-odor and mechanical damage, were used. Upon arrival, visible fat and connective tissue were aseptically removed using sterilized surgical blades in a Class 100 vertical laminar flow cabinet (SW-CJ-1FD, Shanghai Hujing, China). Approximately 60 g of meat was placed in pre-sterilized glassware (250 mL). Six indicator films of 15 mm × 15 mm were affixed to the inner surface of the glassware lid in a circular arrangement (Fig. 5A). All meat samples were then stored in a refrigerator at 4 °C for a duration of six days. Each day, three meat samples were randomly selected for testing. These samples were analyzed for TVB-N, lipid oxidation value and pH value. Concurrently, to mitigate the potential effects of light and humidity, the color parameters of the array were adjusted by subtracting the color parameters of a blank film positioned at the center point of the glassware. The adjusted color parameters were subsequently used to calculate ΔE value throughout the storage period. Given the diverse pigment combinations present in the freshness indicator, the average ΔE value of the films was designated as X.

#### 2.7.2 Determination of thiobarbituric acid reactive substances (TBARS)

The TBA assay was used to evaluate the lipid oxidation of pork samples, as described by Ma and Xiong (2011). In brief, 5 g of pork samples were submerged in 15 mL of 7.5% TCA solution and treated by a homogenizer for 3 min (FSH-2 A, Bona Tech, CHN). Afterward, the homogenate was filtered and then 2 mL of 0.02 mol/L TBA was mixed with the filtrate. Following this, the mixture was heated at 95 °C for 40 min, and then cooled with chilled water. Finally, the absorbance of the resulting solution was assessed at 532 nm using an UV-spectrophotometer (UV-1800PC, AOELAB, China). The results were quantified in terms of mg of malondialdehyde or TBARS per kg of meat, employing the following Eq. (7) for calculation.

(7) $$\text{TBARS}\;\left(\mathrm{mg}/\mathrm{kg}\right)=\frac{\mathrm{Abs}}{W_{s}}\times 9.24$$

where *Abs* was the absorbance of the solution at 532 nm, *W*_*s*_ was the weight of the sample, and 9.24 was a constant derived from the sample dilution and the molar extinction coefficient (156,000 M^−1^·cm^−1^).

#### 2.7.3 pH determination

For the determination of pH value, 20 g of pork samples were chopped and homogenized in 100 mL distilled water for 3 min. Following this, the mixture was placed at 4 °C for 30 min, after which the pH value was assessed by a pH meter.

#### 2.7.4 Measurements of total volatile basic nitrogen (TVB-N)

The TVB-N values of pork tenderloin packaged with the colorimetric array were determined by the Kjeldahl method. In summary, 20 g of pork samples were extracted, diced into pieces, and then placed into a plastic tube. After that, 100 mL of distilled water was introduced and homogenized for 3 min. Following this, the homogenate was incubated at 4 °C for 30 min prior to filtration. Subsequently, a mixture of 5 mL filtrate and 5 mL MgO solution (10 g/L) was added to the Kjeldahl nitrogen tube. Additionally, the boric acid solution, containing 5–6 drops of indicator (10 mL, 20 g/L), was transferred to the receiving bottle. Following a distillation period of 5 min, the solution in the receiving bottle was titrated to the endpoint using a 0.01 M HCl solution. For the blank group, the pork samples were replaced with distilled water. The TVB-N values were reported as mg of nitrogen per 100 g sample (mg/100 g).

### 2.8 Statistical analysis

All measurements were performed at least three times, with results presented as the mean ± standard deviation (SD). All experimental data were analyzed by GraphPad Prism 9 (GraphPad, Boston, USA) and Origin 2022 (OriginLab, Northampton, USA). Analysis of variance and Tukey's multiple comparison test were employed to assess significance at a 95% confidence level using SPSS 27 (IBM, Armonk, USA).

## 3 Results and discussion

### 3.1 Color variations and UV–vis analysis of pigments in different pH solutions

The color variations and UV–vis absorption spectra of pigments across a pH range of 4.0 to 11.0 are illustrated in Fig. 1. From Fig. 1A, it was evident that Cur displayed a light yellow hue at pH values of 6.0 or lower. The increase in pH value was associated with a significant reduction in the yellowness of the pigment solution. At pH of 8.0, the solution approached a colorless state, while at pH values between 9.0 and 11.0, it exhibited an orange hue. Concurrently, the UV absorption spectra of Cur exhibited corresponding changes at varying pH values. These color changes of Cur and shifts in the maximum absorption wavelength could be ascribed to the reversible processes of protonation and deprotonation of Cur molecules under acidic and alkaline conditions (Li et al., 2024). Specifically, under acidic and neutral conditions, the ketone form of Cur predominated, resulting in a light yellow coloration. In contrast, under alkaline conditions, the enol type gradually became dominant, producing an orange or reddish-brown color. The colorimetric alterations were also observed in alizarin solutions at various pH values. In acidic solutions (pH 4.0–6.0), alizarin appeared light yellow. As the pH ranged from 7.0 to 11.0, it transitioned to a bluish-purple color. These color changes can be attributed to the reversible structural transformation of alizarin in response to differing pH values (Chen et al., 2021). At pH values of 4.0 to 6.0, the nitro group, azo group, and phenolic hydroxyl group within the aromatic ring became ionized, resulting in an uncharged molecule characterized by a yellow hue. As the pH continues to rise, the phenolic hydroxyl group dissociated as a result of resonance effects, imparting a purple color to the solution (Ezati et al., 2020). The UV–vis spectra of alizarin under varying pH values (Fig. 1C) further corroborated the pH sensitivity of alizarin. It was apparent from Fig. 1A that the mixed pigments exhibited a broader range of color changes across different pH solutions, particularly between pH 6 and 8. This pH range corresponded to the pH variations observed in pork during the spoilage process, suggesting potential of these pigments as meat freshness indicators.

**Fig. 1:**
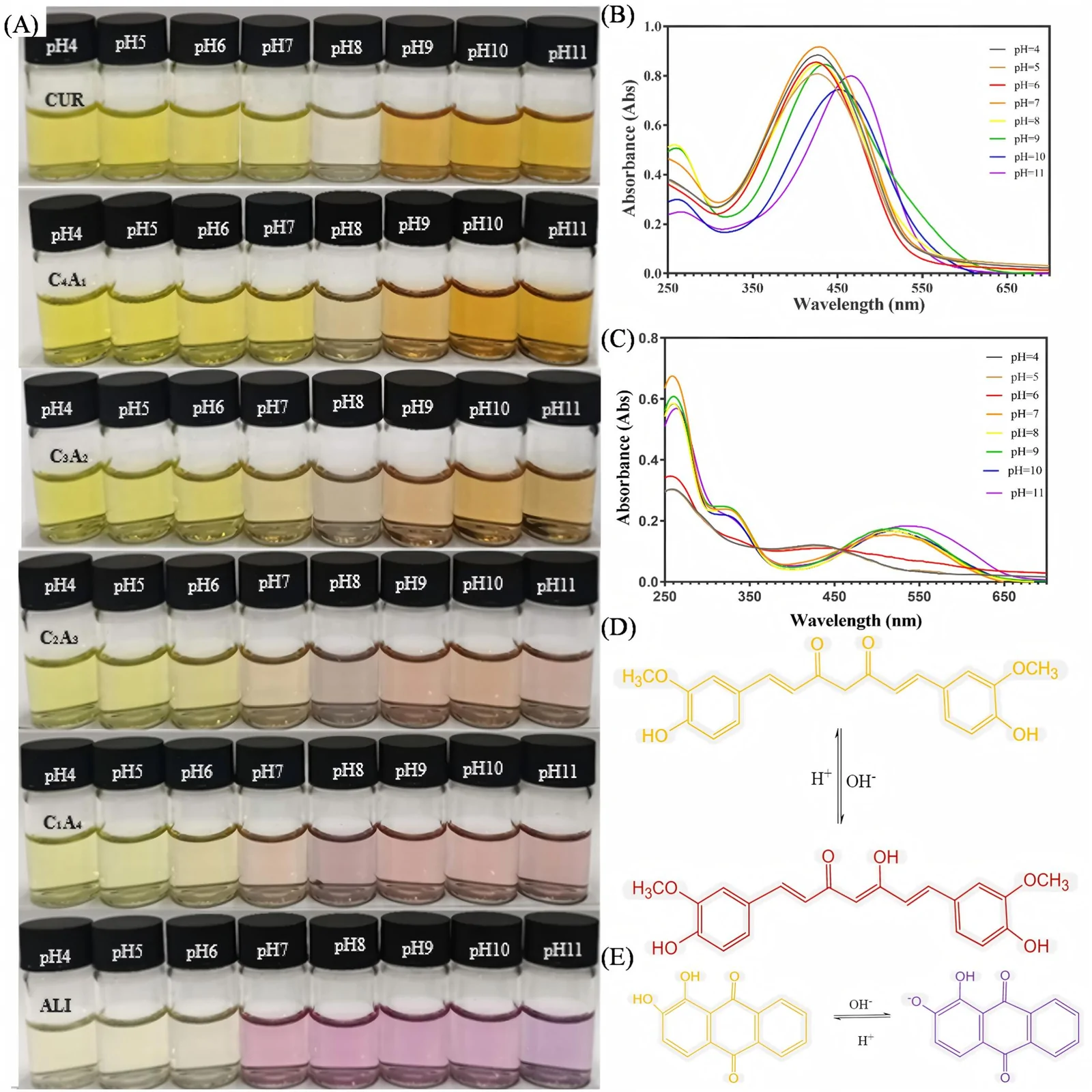
(A) Color responses of different proportions of pigments; (B–C) UV–vis spectra of Cur and Ali in different pH solutions; (D-E) Discoloration mechanism of Cur and Ali.

### 3.2 SEM analysis

As illustrated in Fig. 2A, the PA nanofibers exhibited a generally smooth yet uneven surface, characterized by a notably larger average fiber diameter of 347.44 ± 5.57 nm. This may be attributed to the combined effects of the high viscosity of spinning solution and the entanglement of polymer chains induced by the electrostatic field during the spinning process (da Silva Parize et al., 2016). Upon mixing PVA and zein, a decrease in solution viscosity was observed, leading to a reduction in the diameter of the PZ fibers to 89.39 ± 2.40 nm. This reduction in viscosity aligns with prior studies demonstrating that lower polymer entanglement in mixed solutions produces finer fibers during electrospinning (Deng et al., 2020). This rheological modification enhances jet elongation under electrostatic forces, facilitating the formation of submicron-scale fibrous architectures. From a mechanistic perspective (Amor et al., 2024), the attenuated viscosity reduces the solution's surface energy, thereby minimizing inter-fiber agglomeration during the electrohydrodynamic process. Microscopic analysis revealed continuous cylindrical fibers with smooth surfaces and minimal fusion points, indicative of favorable molecular compatibility between PVA and zein. Additionally, it is noteworthy that the introduction of pigments had minimal impact on the overall morphological characteristics of the nanofibers. However, interesting results about variations in fiber diameter were found based on the specific pigment used. The inclusion of Cur resulted in an increase in fiber diameter to an average of 119.87 ± 1.60 nm. Conversely, the incorporation of alizarin resulted in a reduction of fiber diameter to an average of 75.93 ± 1.60 nm. In addition, the average nanofiber diameters of C_4_A_1_, C_3_ A_2_, C_2_A_3_ and C_1_A_4_ were measured as 104.90 ± 1.59, 92.57 ± 2.15, 83.10 ± 1.60 and 82.27 ± 1.76 nm, respectively. These variations in fiber diameter may be owing to the formation of intramolecular hydrogen bonds between Cur and zein, which increased the viscosity of the spinning solution (Xie et al., 2022). In contrast, the presence of dihydroxyl structure in alizarin appeared to decrease the viscosity of the spinning solution. This structural feature facilitated the creation of hydrogen bonds between molecules.

**Fig. 2:**
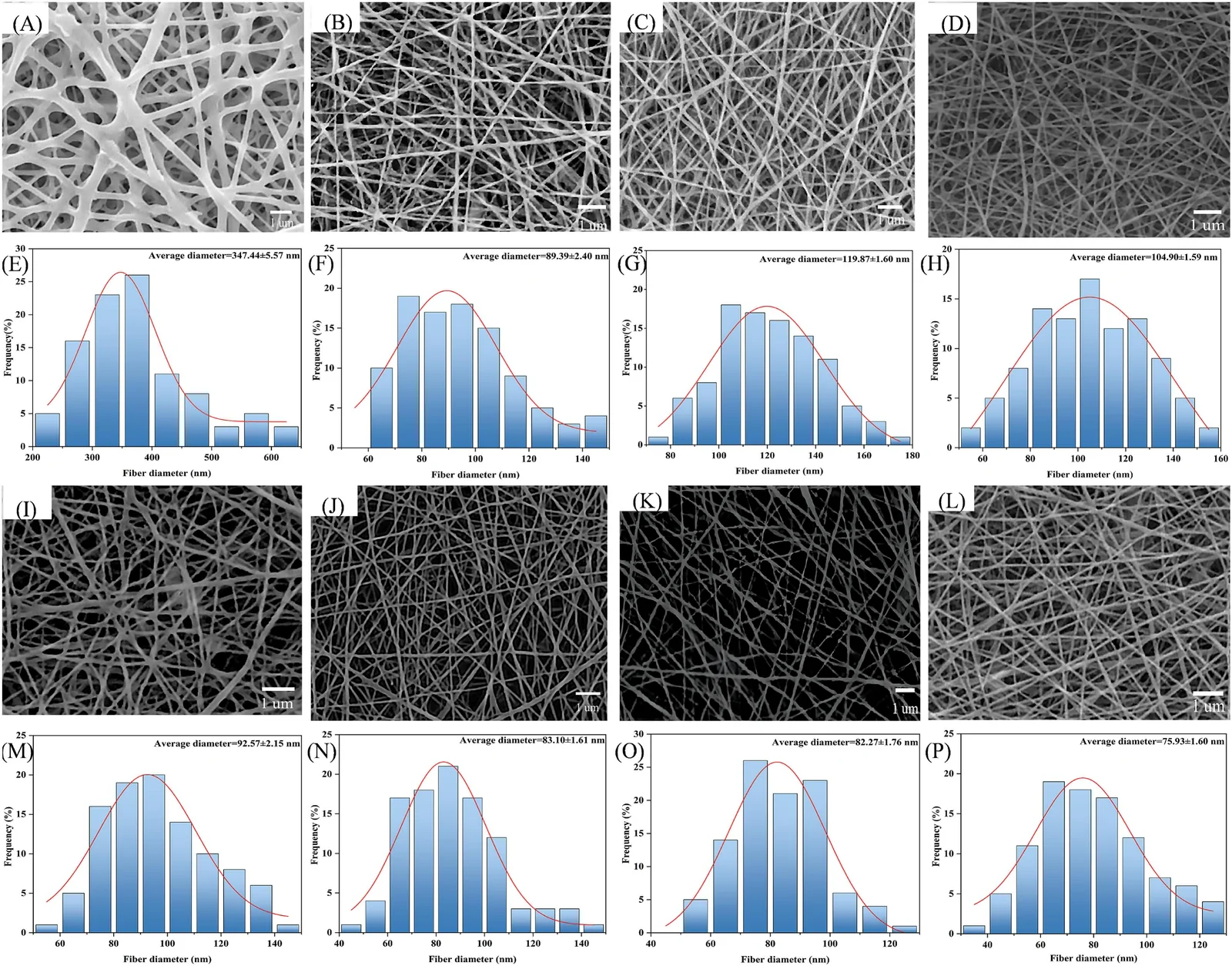
Morphologies of (A) PA, (B) PZ, (C) CR, (D) C_4_A_1_, (I) C_3_A_2_, (J) C_2_A_3_, (K) C_1_A_4_ and (L) AL. Diameter analysis of (E) PA, (F) PZ, (G)CR, (H) C_4_A_1_, (M) C_3_A_2_, (N) C_2_A_3_, (O) C_1_A_4_ and (P) AL.

### 3.3 FTIR analysis

The FTIR analysis of all film formulations was presented in Fig. 3A. On account of the minimal quantity of pigments, the infrared spectrum exhibited no significant alterations compared with PZ. All spectra displayed similar patterns, but with variations in transmittance values. The band at 2940 cm^−1^ was assigned to the asymmetric stretching vibration of C—H in the methylene and methyl groups of the PVA backbone and of the aliphatic side chains of zein, whereas the band at 1092 cm^−1^ was assigned to the C—O stretching vibration of the secondary alcohol groups of PVA (Ali et al., 2019; Kenawy et al., 2018). For all the film samples, a prominent peak at 3200 cm^−1^ was observed, representing of the stretching vibration of O—H groups. The incorporation of zein resulted in enhanced transmittance at the characteristic peaks of zein in comparison to the PA films. For example, characteristic peaks were identified at 1650 cm^−1^ (zein amide I band), 1538 cm^−1^ (zein amide II band) and 1255 cm^−1^ (zein amide III band) (Ren et al., 2022). These peaks were associated with the stretching vibration of C=O (I), the N—H bending vibration (II) and the N—H bending vibration (III), respectively. The results suggested that the pigments were well-compatible with the matrix, forming a homogeneous film through their interactions.

**Fig. 3:**
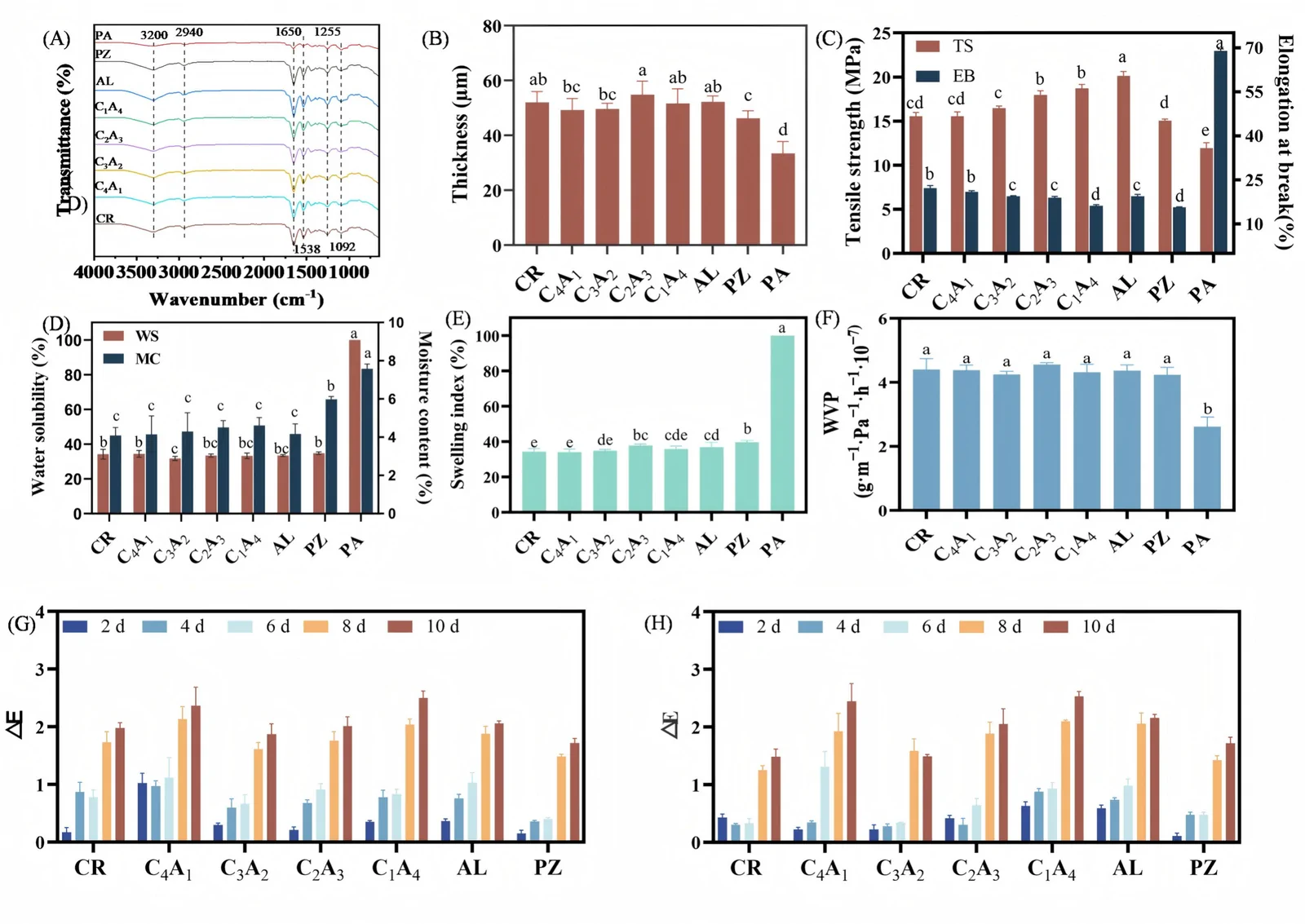
(A) FTIR spectra, (B) Thickness, (C) Tensile strength and elongation at break, (D) Water solubility and moisture content, (E) Swelling index, (H) WVP, (G, H) Temperature stability analysis at 4 °C and 25 °C of PA, PZ, CR, C4A1, C3A2, C2A3, C1A4 and AL. Different lowercase letters indicated significant differences between groups (*P* < 0.05).

### 3.4 Thickness and mechanical properties

For packaging materials, the thickness is a critical factor that affects their mechanical and barrier properties. As illustrated in Fig. 3B, the thickness of each film varied between 30 and 60 μm. Compared with the pure PA film, the incorporation of hydrophobic curcumin into the hydrophilic PVA matrix caused structural discontinuities via interfacial phase separation, resulting in a 40% increase in thickness. In general, the introduction of Cur and alizarin resulted in a notable increase in film thickness, which was consistent with the findings of Cui et al. (2024). Since the total pigment content remained constant, the incorporation of pigments did not notably alter the film thickness. Fig. 3C illustrated the tensile strength (TS) and elongation at break (EB) of the films. PA demonstrated robust TS and excellent EB, similar to those reported previously. As a polymer material with poor mechanical properties, the incorporation of zein significantly decreased EB of PZ, but TS was improved because the formation of hydrogen bonds strengthened the network structure among the nanofibers, as mentioned in the FTIR analysis. Compared with PA, the increased alizarin content enhanced the rigidity of the film but negatively affected its ductility, which was also documented in Zhang's study (Zhang et al., 2024). Furthermore, the diameter and uniformity of the nanofibers may also affect the mechanical strength. Han et al. (2020) reported that a smaller nanofiber diameter correlated with fewer fiber defects and stronger tensile resistance, which was verified by the film morphology.

### 3.5 Water sensitivity

WS, MC and SI were vital indicators for assessing the water sensitivity of packaging materials. These factors provide valuable insights for the potential application of films, particularly in environments with high humidity. As shown in Fig. 3D and E, the pure PVA film exhibited a WS of 100%, an MC of 7.58%, and an SI of 100%, indicating complete dissolution. The addition of zein, Cur and alizarin significantly reduced the WS, MC and SI of PVA-based films (*P* < 0.05). Except for PA, the WS of other films was between 31% and 35%, of which C_3_A_2_ was as low as 31.78%. Meanwhile, the SI and WS demonstrated similar trends. After incorporating zein and pigments, the SI decreased by 35%. When compared to PZ, the water content decreased from 5.98% to 4.5% with varying proportions of Cur and alizarin added. This reduction can be primarily attributed to the intermolecular forces between the hydrophobic material of zein and PVA, which reduced the exposure of the polar side chain groups to the outside, as previously reported in the literature (Giteru et al., 2021). Moreover, molecular interactions between hydroxyl or carboxyl groups within the matrix and hydroxyl or carbonyl groups in the pigments may result in a reduction in the number of active sites accessible for the binding of water molecules (Yang et al., 2024). Therefore, CR, C_4_A_1_, C_3_A_2_, C_2_A_3_, C_1_A_4_ and AL exhibited a superior ability to maintain structural integrity and shape stability in high-humidity environments.

### 3.6 WVP

WVP serves as a vital metric for assessing the moisture resistance of food packaging materials. Generally, lower WVP values are preferred, as they indicate less water loss from packaged food into the surrounding environment. In this study, an intriguing discovery was that while the WVP of PA was 2.62 × 10^−7^ g/(m·Pa·h), the incorporation of zein and pigments significantly increased this value to 4.50 × 10^−7^ g/(m·Pa·h) (*P* < 0.05). This finding contradicted the earlier hypothesis (He et al., 2020) that incorporating hydrophobic materials would decrease the affinity of hydrophilic films for water vapor, thereby enhancing their water vapor barrier performance. The observed phenomenon may be attributed to the significant increase in film thickness resulting from the incorporation of zein and pigments (*P* < 0.05), which exhibited a positive correlation with WVP. In addition, the infiltration of water vapor into the opposite side of the film was mainly composed of two parts: adsorption of water molecules and diffusion to the outward (Yadav et al., 2020). The preceding analysis of micro-morphology indicated that, in comparison to PA, the other films exhibited thinner nanofibers, higher porosity, and a looser network structure, thereby enhancing the diffusion of water molecules. However, given that the colorimetric array was employed within the interior of food packaging and did not directly interface with the external environment, its barrier performance adequately met the requirements for packaging materials. Therefore, this characteristic had minimal impact on the application of freshness index.

### 3.7 Temperature stability

Natural pigments like Cur and alizarin, which contain unsaturated bonds, are sensitive to changes in the external environment, causing color loss in practical applications (Molina et al., 2023). In this study, the films were stored at 4 °C and 25 °C, respectively, to investigate whether the storage environment adversely affected their color stability. As illustrated in Fig. 3G and H, ΔE values for all films increased with extended storage duration. This alteration may be ascribed to variations in relative humidity between the storage conditions and the experimental environment (Jiang et al., 2024). Compared with PZ, the color changes of CR, C_4_A_1_, C_3_A_2_, C_2_A_3_, C_1_A_4_ and AL were smaller, with some even lower than those of PZ. This indicated that Cur and alizarin were effectively encapsulated within the film matrix. Overall, the ΔE values of the films remained below 3 during storage, while it was generally accepted that a ΔE greater than 5 was perceptible to untrained observers (Prietto et al., 2017). Therefore, it could be concluded that CR, C_4_A_1_, C_3_A_2_, C_2_A_3_, C_1_A_4_ and AL exhibited sufficient color stability for use as meat freshness indicators in subsequent stages.

### 3.8 Sensitivity to volatile amines

The colorimetric response of the films to volatile amines produced during meat spoilage was investigated through a variety of experiments aimed at assessing the array sensitivity to different amines. Fig. 4 illustrated the color variations and differences of films exposed to varying concentrations of volatile amine vapors (1, 5, 10, 20, and 40 ppm) as well as different amines, including ammonia, DMA, and TMA. The color alterations of the indicator labels were attributed to the transformation of Cur and alizarin structures with the changes in environmental pH values. The results indicated that an increase in reactant concentration was associated with more pronounced alterations in the color of the films. In general, the color change of AL was not obvious during the experiment, whereas CR exhibited a notable color response. Using ΔE > 5 as an indicator for color change in ammonia solution, the detection limits for CR, C_4_A_1_, C_3_A_2_ and C_2_A_3_ were found to be as low as 1 ppm, while those for C_1_A_4_ and AL were 5 ppm. In dimethylamine solution, CR, C_4_A_1_, C_3_A_2_, C_2_A_3_, and C_1_A_4_ exhibited strong color changes at a concentration of 1 ppm, but AL demonstrated only a weak color change. For CR in trimethylamine solution, the minimum detection range was between 10 and 20 ppm, whereas color changes in C_4_A_1_, C_3_A_2_, C_2_A_3_, C_1_A_4_ and AL occurred at a concentration of 10 ppm. When compared to CR film, the combination of mixed pigments demonstrated enhanced sensitivity in trimethylamine solutions. Similarly, compared to AL film, mixed pigments exhibited improved sensitivity in ammonia and dimethylamine environments. Overall, the use of mixed pigments broadened the color variation range, resulting in higher sensitivity and more pronounced color changes. Among the tested samples, C_4_A_1_, C_3_A_2_, and C_2_A_3_ exhibited the most noticeable color changes. Furthermore, Moeinpour's study (Moeinpour & Mohseni-Shahri, 2023) on freshness indices prepared by mixing alizarin and anthocyanins supported this finding, as the pigment mixture yielded a greater color variation range compared with alizarin alone.

**Fig. 4:**
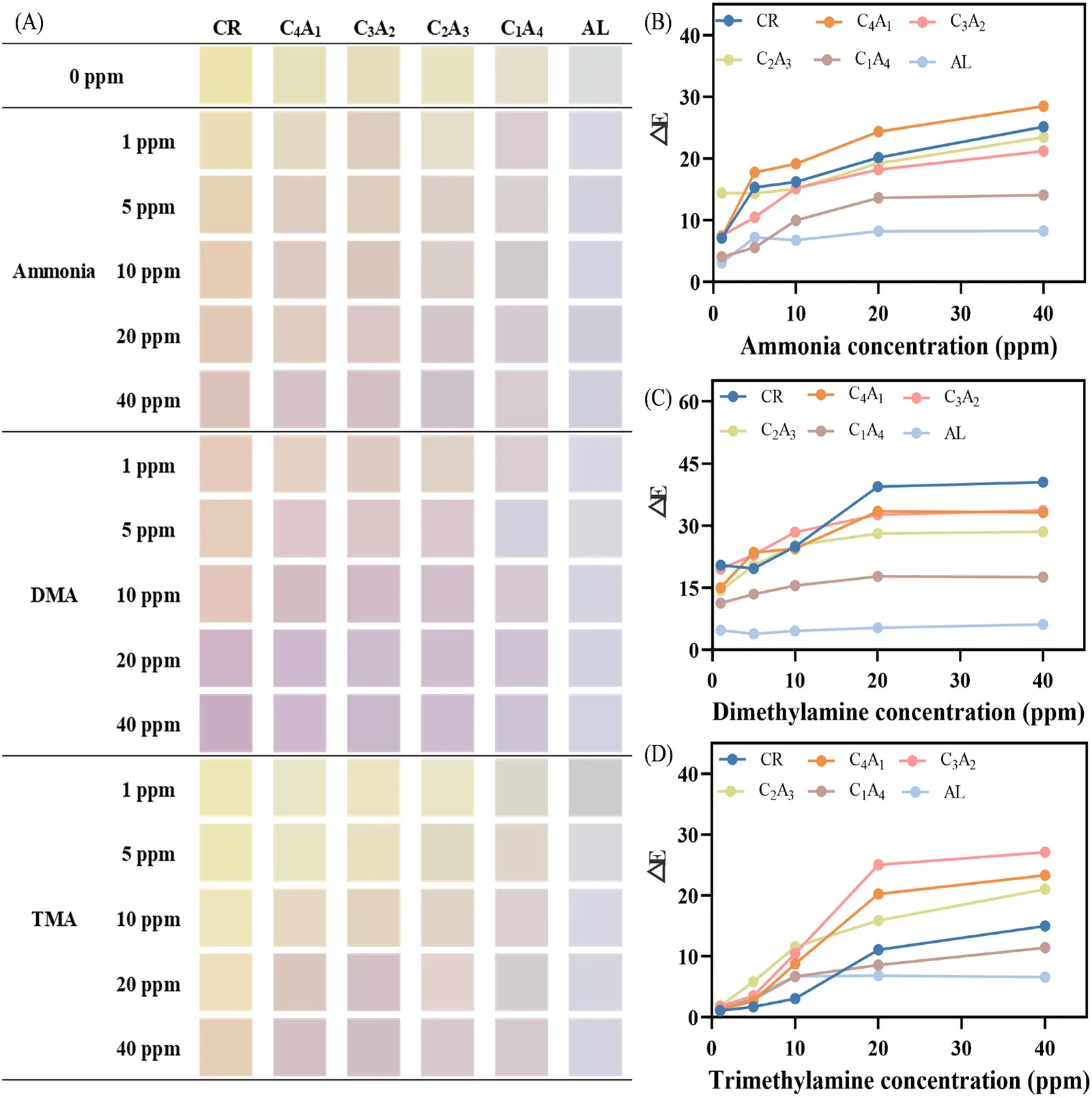
Colorimetric response of the nanofiber films to volatile amines. (A) Digital photographs of CR, C_4_A_1_, C_3_A_2_, C_2_A_3_, C_1_A_4_ and AL films after exposure to ammonia, DMA and TMA vapors at 1, 5, 10, 20 and 40 ppm for 20 min in a sealed reaction chamber. (B–D) Total color difference (ΔE) of the films as a function of amine concentration for (B) ammonia, (C) DMA and (D) TMA under the same conditions.

### 3.9 Pork freshness monitoring

#### 3.9.1 TBARS, pH and TVB-N changes in tenderloin

TBARS, pH, and TVB-N are three important indices for evaluating the spoilage of fresh meat. TBARS assessed the extent of lipid oxidation in meat, while pH and TVB-N reflected the degree of nitrogenous compound decomposition. As illustrated in Fig. 5E, the TBARS value of pork commenced at 0.16 ± 0.01 mg/kg, and exhibited an upward trend with prolonged storage duration, ultimately attaining a value of 0.52 ± 0.02 mg/kg on day 4. Although there was no universally accepted limit for the TBARS value, it was generally believed that when the value exceeded 0.5 mg/kg, a peculiar odor would develop during processing. The pH value of pork exhibited an initial decline followed by an increase throughout the storage period, dropping from 5.96 ± 0.03 to 5.87 ± 0.02 before ultimately rising to 6.58 ± 0.02. The initial decrease in pH was caused by lactic acid produced from glycogen degradation and phosphoric acid generated from the degradation of adenosine triphosphate (Bekhit et al., 2021). As glycogen was continuously consumed, glycogen-dependent microorganisms gradually transitioned to protein-degrading bacteria. This transition promoted the decomposition of amino acids and proteins, resulting in the generation of alkaline ammonia compounds and an increase in the pH value. According to the National Standard GB 2707-2016, the TVB-N content in fresh meat should not exceed 15 mg/100 g. The analysis of TVB-N values revealed that the samples' values were below 10 mg/100 g on day 1, indicating that the pork was still fresh. Subsequently, on days 2 and 3, the TVB-N values ranged from 10 to 15 mg/100 g, suggesting that the pork samples had entered to a less fresh state. The onset of spoilage was observed on day 4, with a TVB-N value of 15.54 ± 0.39 mg/100 g. By integrating the experimental findings from TBARS, pH, and TVB-N, the tenderloin was categorized as fresh on the 1st day, less fresh on the 2nd and 3rd days, spoiled by the 4th day, and deemed completely unacceptable after the 6th day.

**Fig. 5:**
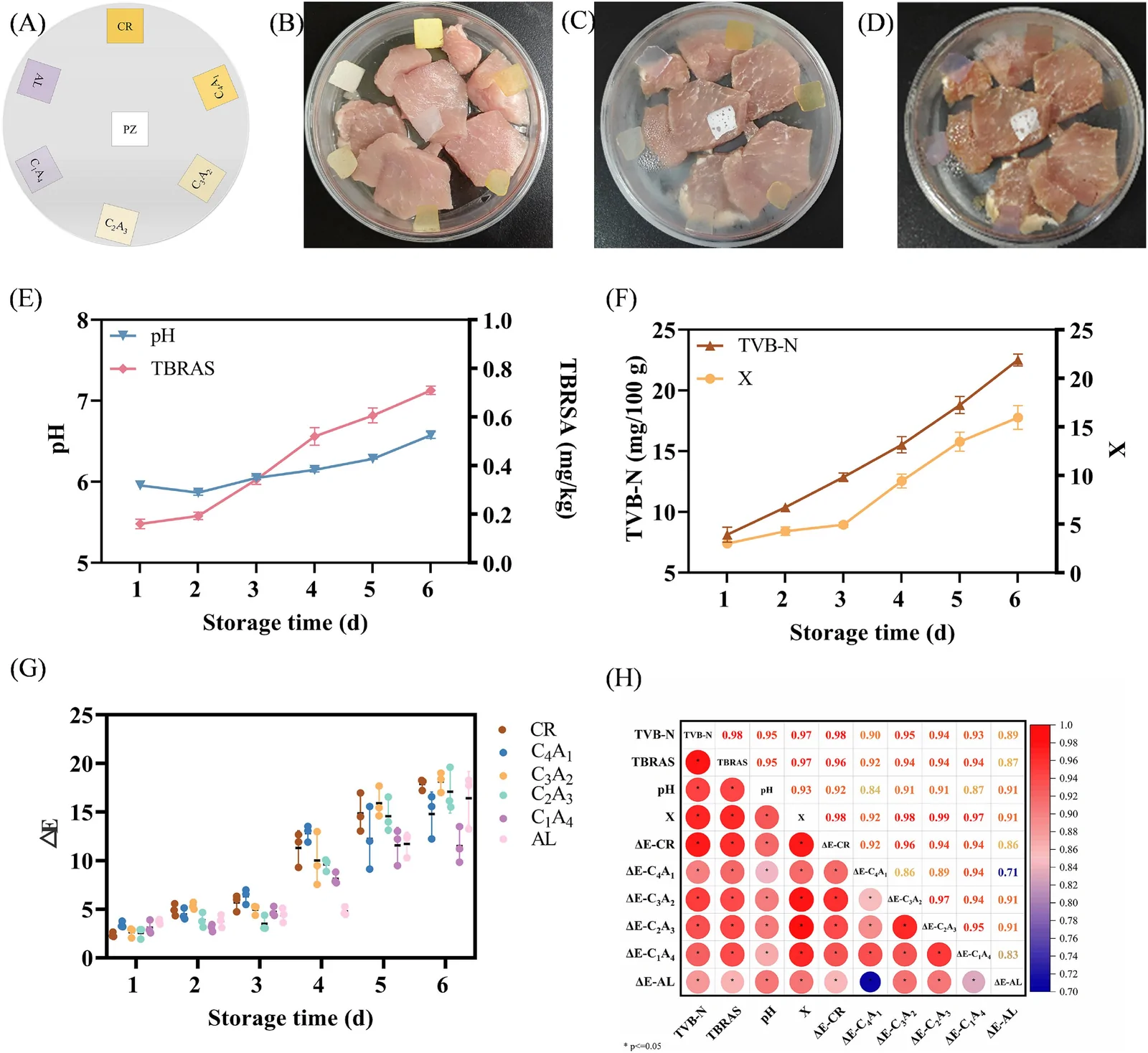
(A) Diagram of the location of the indicating array; Freshness monitoring of pork tenderloin: (B) fresh, (C) less fresh and (D) spoiled; Application of colorimetric array in the freshness monitoring: (E) the pH and TBARS values, (F) the TVB-N and X values, (G) the △E changes of CR, C_4_A_1_, C_3_A_2_, C_2_A_3_, C_1_A_4_ and ALI, (H) Pearson correlation coefficient heat map.

#### 3.9.2 Color changes of indicator labels for tenderloin spoilage trial

The prepared films (smart film and control), serving as labels on the packaging, were affixed to the interior of the meat-containing packages to monitor reactions to volatilization during meat decay. Fig. 5B-D illustrated the observed color variations of the indicator films throughout the storage period. The indicator array gradually evolved from light yellow to reddish-brown and purple, while the mixed pigment label displayed a series of transitional colors. In Fig. 5F, the chromatic parameter X initially increased from 3.00 ± 0.03 to 4.95 ± 0.14 on the 3rd day, and then increased sharply to 9.44 ± 0.41 on the 4th day. At this point, obvious color alterations were discernible to the unaided eye. Compared with the colorimetric array, the color changes of a single indicator tag, such as AL or C_1_A_4_, were often delayed or not very noticeable in practical applications. The colorimetric array effectively compensated for this limitation by processing and analyzing a variety of film information. The Pearson coefficients between the X value and TBARS, pH, TVB-N were 0.98, 0.95, and 0.98, respectively. The color alterations of the array demonstrated a strong correlation with the spoilage indicators of pork tenderloin (Wu et al., 2026). The color change of the indicator film was highly consistent with the spoilage indices of pork tenderloin, indicating that the colorimetric array could effectively monitor meat product freshness.

## 4 Conclusions

A synergistic PVA/zein electrospun nanofiber network embedded with curcumin and alizarin was successfully fabricated as a pH-sensitive colorimetric indicator array. The water solubility, moisture content, and swelling index of the films decreased from 100.00%, 7.58% and 100.00% (pure PVA film) to 31.74%–35.42%, 4.50%–5.98% and 65.00% (*P* < 0.05). The PVA/zein composite achieved exceptional water resistance through synergistic hydrophobic and hydrogen-bonding interactions, significantly improving structural stability compared to pure PVA film, while the continuous nanofibrous structure (75.93 ± 1.59–119.87 ± 1.60 nm) and the color stability (ΔE < 3 after 10 d at 4 and 25 °C) were maintained. The curcumin-alizarin dual-pigment system enabled full-range pH response and ultra-high sensitivity, with a 1 ppm detection limit for ammonia and dimethylamine vapors, five times lower than that of the alizarin-only film. The color changes of the array strongly correlated with pork spoilage markers, including TVB-N (*r* = 0.98), TBARS (*r* = 0.98), and pH (*r* = 0.95), supporting accurate freshness assessment. A visual three-tier grading system was established for user-friendly on-site monitoring. This eco-friendly and scalable platform advances intelligent packaging technologies, providing a low-cost, practical solution for real-time food quality visualization and holding great promise for food safety applications.

## CRediT authorship contribution statement

**Bingcan Liu:** Writing – original draft, Methodology, Investigation, Formal analysis. **Siling Zhang:** Writing – original draft, Methodology, Investigation, Formal analysis. **Honghui Geng:** Methodology, Investigation. **Can Hu:** Supervision, Resources, Methodology. **An Zhou:** Resources, Methodology. **Zeyu Wu:** Writing – review & editing, Supervision, Funding acquisition, Data curation, Conceptualization.

## Declaration of competing interest

The authors declare that they have no known competing financial interests or personal relationships that could have appeared to influence the work reported in this paper.

## Data Availability

Data will be made available on request.
